# TNF-α Quantification in Formalin-Fixed Paraffin-Embedded Tissues as a Predictive Biomarker in Ulcerative Colitis

**DOI:** 10.3390/diagnostics15232946

**Published:** 2025-11-21

**Authors:** Anna Viola, Walter Giuseppe Giordano, Rasmus Goll, Emanuela Germanà, Vincenzo Fiorentino, Valeria Zuccalà, Gabriele Ricciardi, Mariagiovanna Ballato, Pietro Tralongo, Antonio Ieni, Guido Fadda, Giuseppe Giuffrè, Maurizio Martini, Walter Fries

**Affiliations:** 1 Department of Clinical and Experimental Medicine, University of Messina, 98124 Messina, Italy; aviola@unime.it (A.V.); walter.fries@unime.it (W.F.); 2 Department of Biomedical, Dental and Morphological and Functional Imaging Sciences, University of Messina, 98124 Messina, Italy; waltergiordano1997g@gmail.com (W.G.G.); emanuelagermana@hotmail.it (E.G.); gricciardi1998@gmail.com (G.R.); mariagiovannaballato96@gmail.com (M.B.); pietrotralongo@gmail.com (P.T.); 3 Division of Gastroenterology, Department of Internal Medicine, University Hospital of North Norway, 9019 Tromsø, Norway; rasmus.goll@uit.no; 4 Department of Human Pathology of the Adulthood and Developing Age “Gaetano Barresi”, Section of Pathology, University of Messina, 98125 Messina, Italy; vincenzo.fiorentino@unime.it (V.F.); valeria.zuccala@unime.it (V.Z.); antonio.ieni@unime.it (A.I.); guido.fadda@unime.it (G.F.); giuseppe.giuffre@unime.it (G.G.)

**Keywords:** IBD, ulcerative colitis, TNF-α, biomarker, personalized medicine

## Abstract

**Objectives:** Anti-TNF-α therapies have transformed the management of Inflammatory Bowel Disease (IBD), yet a substantial proportion of patients fail to respond, highlighting the urgent need for predictive biomarkers. Mucosal TNF-α mRNA quantification in fresh biopsies has shown promise but showed several problems for routine clinical use. This study aimed to validate a clinically feasible method for TNF-α quantification in formalin-fixed paraffin-embedded (FFPE) tissues and to assess its correlation with established measures of disease activity. **Methods:** FFPE and matched fresh-frozen biopsies from 54 ulcerative colitis patients were analyzed. Total RNA was extracted from FFPE sections, and TNF-α RNA was quantified by RT-qPCR and compared with fresh tissue expression levels. Molecular data were correlated with clinical (pMayo), endoscopic (Mayo Endoscopic Score, MES), and histological (Geboes) indices. **Results:** TNF-α expression in FFPE samples strongly correlated with fresh tissue levels (r = 0.83, *p* < 0.0001). High TNF-α expression in FFPE tissue was significantly associated with active endoscopic mucosal disease (MES ≥ 1; OR 28, 95% CI 3.31–237; *p* < 0.0001) and with histological inflammation (Geboes ≥ 3.1; OR 0.12, 95% CI 0.02–0.59; *p* = 0.009). Fresh tissue TNF-α levels showed similar associations. Clinical parameters such as age, sex, and pMayo score did not significantly correlate with mucosal TNF-α expression. RT-qPCR quantification of TNF-α in FFPE tissue is a reliable, cost-effective surrogate for fresh biopsy analysis and correlates strongly with endoscopic and histological disease activity. **Conclusions:** This method offers a practical approach for integrating molecular biomarkers into routine pathology workflows, supporting the implementation of personalized treatment strategies in IBD.

## 1. Introduction

Inflammatory Bowel Disease (IBD), encompassing Ulcerative Colitis (UC) and Crohn’s Disease (CD), represents a group of chronic, relapsing-remitting inflammatory disorders of the gastrointestinal tract [1]. The global burden of IBD has been rising steadily, not only in Western countries but also in newly industrialized nations, posing a significant challenge to healthcare systems worldwide [2]. In Italy, the prevalence of ulcerative colitis has steadily increased over the past decades, mirroring the trends observed across other Western European countries. Recent population-based studies have estimated a prevalence of approximately 200–250 cases per 100,000 inhabitants, with incidence rates ranging from 6 to 10 new cases per 100,000 persons per year [3,4]. Data from Northern and Southern Italian regions suggest comparable epidemiological patterns, confirming that ulcerative colitis represents a significant and growing health concern nationwide, including in Sicily, where this study was conducted [3,4].

The pathophysiology of IBD is complex and is thought to result from a dysregulated immune response to intestinal microbes in genetically susceptible individuals [5]. A central player in the inflammatory cascade that drives mucosal damage in IBD is Tumor Necrosis Factor-alpha (TNF-α), a potent pro-inflammatory cytokine [6].

The pivotal role of TNF-α led to the development of biological agents designed to neutralize its activity, such as the monoclonal antibodies infliximab and adalimumab [7]. The introduction of these anti-TNF-α therapies has profoundly transformed the management of moderate-to-severe IBD, leading to higher rates of clinical remission, mucosal healing, and a reduction in hospitalizations and surgeries [8]. Despite their success, the utility of these powerful agents is hampered by significant variability in patient response [8]. Approximately 10–30% of patients exhibit primary non-response, failing to benefit from initial induction therapy, while another 23–46% experience a secondary loss of response over time, necessitating dose adjustments or a switch to a different therapeutic class [9].

This “one-size-fits-all” approach to prescribing anti-TNF-α agents is suboptimal because it exposes non-responsive patients to the potential side effects and high costs of ineffective treatment, while delaying the initiation of a potentially more beneficial therapy [10]. Consequently, a paramount unmet need in the field of IBD is the identification and validation of biomarkers that can accurately predict treatment response before therapy is initiated [11]. An ideal biomarker would allow clinicians to stratify patients, selecting those most likely to benefit from anti-TNF-α therapy, while directing others toward treatments with alternative mechanisms of action, such as those targeting the IL-12/23 pathway or leukocyte trafficking [11].

Current tools for assessing disease activity and guiding treatment decisions, such as clinical activity indices (e.g., partial Mayo score), serum C-reactive protein (CRP), and fecal calprotectin, often correlate poorly with the actual extent of mucosal inflammation [12]. Endoscopy with biopsy remains the gold standard for evaluating mucosal healing, a key therapeutic goal linked to improved long-term outcomes [13]. Measuring the expression of TNF-α mRNA directly within mucosal tissue offers a biologically intuitive approach to patient stratification. It quantifies the abundance of the very molecule the therapy is designed to target. Previous research has provided strong evidence supporting this concept. Studies by Olsen et al. demonstrated that high pre-treatment levels of TNF-α gene expression in fresh colorectal mucosa were an independent predictive factor for clinical and endoscopic remission following induction therapy with infliximab in patients with UC [14]. More recent work by James et al. further explored this relationship, evaluating tissue-specific TNF mRNA with RNAscope in situ hybridization and finding higher expression levels in adult patients and those with primary treatment failure [15,16,17]. These studies collectively hypothesize that patients with a high “TNF-α burden” are the most suitable candidates for anti-TNF-α therapy, and may even require higher doses for effective target engagement [18].

A significant barrier to the widespread clinical adoption of this promising strategy is a practical one. Many of these foundational studies have relied on the analysis of fresh or fresh-frozen intestinal biopsies. This requires specialized collection and storage protocols (e.g., immediate snap-freezing in liquid nitrogen or stabilization in RNAlater), which are not part of standard clinical workflows [19]. These procedures add logistical complexity, increase costs, and are often reserved for research settings. In contrast, formalin-fixed, paraffin-embedded (FFPE) tissue blocks are the universal standard for histopathological diagnosis in gastroenterology. Biopsies are routinely collected and processed into FFPE blocks, and these archives represent a vast, invaluable resource for translational research and potential clinical testing [19].

For years, RNA analysis from FFPE tissue was considered unreliable due to RNA degradation and chemical modification caused by the formalin fixation process [20]. However, recent advancements in RNA extraction kits and sensitive detection technologies, such as real-time quantitative PCR (RT-qPCR), have largely overcome these challenges, enabling robust and reproducible gene expression analysis from archived tissues [21,22].

This study was therefore designed to bridge the gap between the research-grade utility and the clinical feasibility of mucosal TNF-α quantification. Our primary objective was to validate an RT-qPCR-based method for measuring TNF-α mRNA expression in standard FFPE biopsy samples from IBD patients. To achieve this, we aimed to: (1) directly compare the TNF-α expression levels obtained from FFPE tissues with those from matched fresh-frozen samples from the same patient cohort, which served as the reference standard; and (2) correlate the FFPE-derived TNF-α expression levels with established and validated demographic, clinical, endoscopic, and histological scores of disease activity. By validating this more practical and scalable method, we hope to pave the way for its use as a decision-making tool in the clinical management of IBD, facilitating a more personalized and effective therapeutic approach.

## 2. Materials and Methods

### 2.1. Study Design, Patient Cohort and Ethical Considerations

This retrospective cohort study was conducted by analyzing data and biological samples from 54 patients with a confirmed diagnosis of Ulcerative Colitis who were being followed at the Clinical Unit of Gastroenterology of the University di Messina, Italy. All procedures contributing to this work were conducted in accordance with the principles of the Declaration of Helsinki and Good Clinical Practice (GCP) guidelines. The study protocol was formally reviewed and approved by the Ethics Committee of the University of Messina (Prot. 81/2018) on 13 December 2018. All patient data were collected anonymously, and no identifying images or personal or clinical details that could compromise anonymity are included in this report. Patient data and biological samples were collected following protocols established by a collaborative research group including national and international institutions. For the primary analysis, FFPE tissue blocks and corresponding clinical data were utilized. These were matched to pre-existing data from fresh-frozen tissue analyses from the same endoscopic procedure for each patient.

Demographic and extensive clinical data were meticulously extracted from patient medical records. The collected patient characteristics are presented in Table 1. Inclusion criteria for the study were: adult patients (age ≥ 18 years) with an established diagnosis of UC based on standard clinical, endoscopic, and histological criteria, and for whom both fresh-frozen and FFPE mucosal biopsies were available from the same procedure. Patients were recruited between 2018 and 2021. The patient population included individuals on various treatment regimens at the time of sampling, including 5-aminosalicylic acid (5ASA), biological drugs (primarily anti-TNF-α agents), alone or in combination therapy, as well as a small number of treatment-naïve patients (6 out of 54, 11.1%).

### 2.2. Clinical, Endoscopic and Histological Data Collection

Clinical disease activity was systematically quantified using the partial Mayo score (pMS) [23]. The pMS is a widely used and validated non-invasive index composed of two patient-reported components: stool frequency and rectal bleeding. For analytical purposes, patients were stratified into two groups: those with a pMS of 2 or less, representing clinical remission or low disease activity, and those with a pMS greater than 2, indicative of moderate to severe clinically active disease.

All patients included in the study had undergone a full colonoscopy for either diagnostic or surveillance purposes. Endoscopic disease activity was graded at the time of the procedure by the performing endoscopist using the Endoscopic Mayo Score, a standard four-point scale (0–3) [24]. A score of 0 was assigned to mucosa with a normal appearance or inactive disease. A score of 1 reflected mild disease, characterized by erythema, reduced vascular pattern, and slight friability. Moderate disease corresponded to a score of 2, defined by pronounced erythema, complete loss of vascular pattern, friability, and superficial erosions. Severe disease was graded as 3, indicating spontaneous bleeding and the presence of ulcerations. For analytical purposes, patients were categorized into two groups: those achieving mucosal healing (MH), defined as an Endoscopic Mayo Score of 0 or 1, and those with ongoing endoscopic activity, defined by scores of 2 or 3. Histological inflammation was evaluated on hematoxylin and eosin (H&E) stained slides from the FFPE biopsies by a pathologist blinded to the clinical and molecular data. The Geboes score is a detailed histological grading system for UC, with scores categorized as inactive (<3.1) versus active (≥3.1) inflammation [25].

### 2.3. Fresh and FFPE Tissue TNF-α Expression Levels

Colonoscopy was performed up to the terminal ileum; in all the explored sections, at least two biopsy samples were taken according to guidelines. In patients with a new UC diagnosis or with endoscopic disease activity, biopsies were taken from the most inflamed site. In those patients with mucosal healing, biopsies were taken from the previously most inflamed site. For a subset of 30 UC patients, paired biopsies were taken from the same site. One set of samples was immediately snap-frozen in RNA later (Thermofisher Scientific, Waltham, MA, USA) and stored at 4 °C overnight before being stored at −80 °C until analysis. The second set of samples was processed according to standard histopathological laboratory protocol: they were fixed in 10% neutral-buffered formalin for 24 h and then processed through graded alcohols and xylene before being embedded in paraffin wax to create FFPE blocks. These FFPE blocks, along with those from the entire 54-patient cohort, were archived and served as the primary material for this investigation.

### 2.4. RNA Extraction from Formalin-Fixed Paraffin-Embedded (FFPE) Tissues

Total RNA was carefully isolated from the archived FFPE tissue blocks. A microtome was then used to cut five to ten 10 µm-thick sections, which were placed into an RNase-free microcentrifuge tube. The extraction was performed using the RNeasy FFPE Kit (Qiagen, Milan, Italy) in strict accordance with the manufacturer’s instructions. This protocol begins with deparaffinization using xylene to dissolve the paraffin wax. The tissue is then rehydrated and subjected to a robust lysis buffer containing proteinase K. The lysate was then applied to a silica-membrane RNeasy MinElute spin column, where the RNA binds. Following a series of washes to remove contaminants, the purified total RNA was eluted in a small volume of RNase-free water.

### 2.5. Reverse Transcription and Real-Time Quantitative PCR (RT-qPCR) for TNF-α RNA

The relative level of TNF-α RNA transcripts was determined using a two-step RT-qPCR assay (GoTaq Probe 2-step RT-qPCR; Promega, Milan, Italy). In the first step, RNA from each sample was converted into complementary DNA (cDNA) using a high-capacity cDNA reverse transcription kit. In the second step, the qPCR was performed to amplify and quantify the target (TNF-α) and reference (β-actin, ACTB) genes. The amplification reaction was carried out on a CFX96 Real-Time PCR Detection System (Bio-Rad Laboratories, Milan, Italy). This probe-based chemistry employs gene-specific primers and a TaqMan probe with a 5′ reporter dye and a 3′ quencher, which provides high specificity and a strong signal-to-noise ratio. The thermal cycling conditions were optimized for the primers and probes used. The expression of TNF-α was normalized to the expression of β-actin, a stably expressed housekeeping gene, to control variations in the amount and quality of input RNA and the efficiency of the reverse transcription reaction. The primers used were TNF-α S 5′-CAC GCT TCT TCT GCC TGC TG; TNF-α AS 5′-GAT GAT CTG ACT GCC TGG GC-3′; β-actin S 5′-TGC CGA CAG GAT GCA GAA G-3′; β-actin AS 5′-GCC GAT CCA CAC GGA GTA CT-3′. Tre probes used were TNF-α-probe 5′-[FAM] CCA GAG GGA AGA GTT CCC CAG GGA C [BHQ1]-3′; β-actin-probe 5′-[HEX] AGA TCA AGA TCA TTG CTC CTC CTG AGC GC [BHQ2]-3′.

Relative gene expression of TNFα was calculated using the 2^−ΔΔCt^ method, and the expression data were normalized with endogenous β-actin. For categorical analysis, a cut-off value of 0.67 (the median expression value in the cohort) was used to dichotomize patients into a “high expression” group and a “low expression” group.

### 2.6. Fresh Tissue TNF-α RNA Expression

Colon tissue biopsies were disrupted using a Tissue Ruptor Disposable Probe (Qiagen, Hilden, Germany). RNA was isolated with the RNeasy Micro Kit (Qiagen) according to the manufacturer’s guidelines. One microgram of the extracted RNA was then reverse-transcribed in a final volume of 20 μL using the QuantiTect Reverse Transcription Kit (Qiagen). Quantitative PCR analyses were carried out on a CFX96 Real-Time PCR System (BioRad, Hercules, CA, USA) employing the NovaPrima TNF qPCR kit (NovaTec, Dietzenbach, Germany). Amplification was initiated with a denaturation step at 95 °C for 3 min, followed by 44 cycles consisting of 95 °C for 10 s and 60 °C for 30 s. Each assay used 2 μL of cDNA as input, and all measurements were performed in duplicate. Quantification was based on a standard curve provided by the kit, and results were normalized against the reference gene RNA polymerase subunit II A (POLR2A). Normalization was applied at the cycle threshold (CT) level using the geometric mean of POLR2A across the plate. Final TNF-α expression levels were expressed as copies per microgram of total RNA. The TNF-α mRNA levels from fresh biopsies were quantified using the NovaPrime TNF kit (NovaTec, Dietzenbach, Germany), following the manufacturer’s protocol, with results expressed in absolute terms as copies per nanogram of RNA. For categorical analysis, a cut-off value of 3174 (the median level value in the cohort) was used to dichotomize patients into a “high expression” group and a “low expression” group.

### 2.7. Statistical Analysis

All statistical computations were performed using GraphPad Prism version 8.0 (GraphPad Software, San Diego, CA, USA) and SPSS version 25 (IBM Corp., Armonk, NY, USA) [26]. Continuous data were presented as mean and standard deviation (SD). Categorical data were presented as numbers and percentages. To address the primary study objective, the relationship between TNF-α expression measured in fresh tissue (copies/ng) and that measured in FFPE tissue (relative expression via the 2^−ΔΔCt^ method) was assessed using Pearson correlation analysis after confirming a linear relationship. The result was visualized with a linear regression plot. To address the secondary objective, we used Fisher’s exact test. Odds ratios (OR) with their corresponding 95% confidence intervals (CI) were calculated to estimate the magnitude and direction of these associations. For all statistical tests performed, a *p*-value of less than 0.05 was considered to indicate statistical significance.

## 3. Results

### 3.1. Patient Characteristics

The study cohort comprised 54 patients with IBD. The baseline demographic, clinical, and biomarker characteristics of the population are detailed in Table 1. The mean age of the cohort was 50 years (SD ± 15.35), with a slight majority of patients (*n* = 28, 51.9%) being aged 50 or older. The cohort was also balanced with respect to sex, with 29 males (53.8%) and 25 females (46.2%). The mean disease duration of the cohort was 9.6 years (SD ± 9.2). Thirty patients had a left-sided colitis (55.6%), 18 out of 54 had a pancolitis (33.3%), while only 6 patients had a proctitis (11.1%).

**Figure 1:**
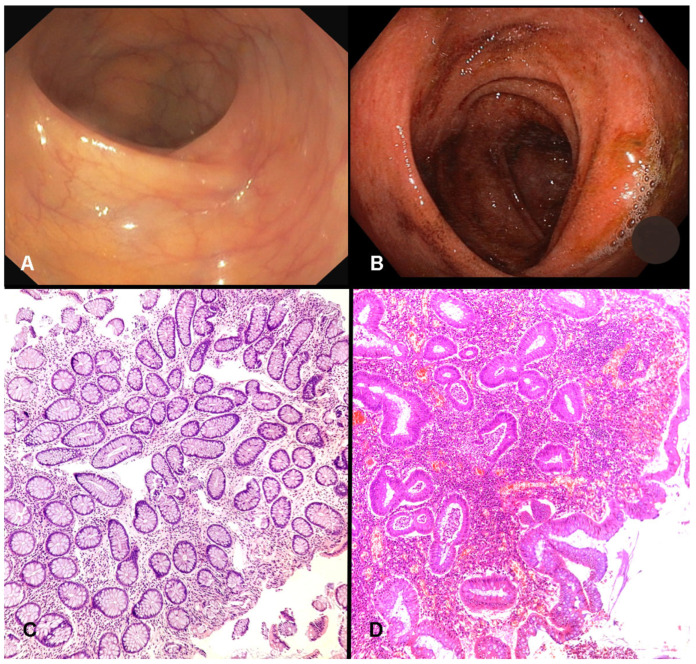
Endoscopic and histologic assessment of disease activity. (**A**) Endoscopic view consistent with Mayo 0, showing normal colonic mucosa without signs of inflammation. (**B**) Endoscopic appearance corresponding to Mayo 2, characterized by marked erythema, loss of vascular pattern, and friability. (**C**) Histologic section compatible with Geboes 0.2, demonstrating low architectural distortion with chronic inflammatory infiltrate but without intraepithelial neutrophils (×10 magnification). (**D**) Histologic section consistent with Geboes 3.2, showing active inflammation with marked presence of neutrophils in colonic epithelium and crypt abscesses (×10 magnification).

At the time of sampling, clinical and endoscopic assessments revealed a population with a broad spectrum of disease activity. According to the Mayo Endoscopic Score (MES), 39 patients (72.2%) showed endoscopic activity (MES ≥ 1), while 15 (27.8%) achieved mucosal healing (MES < 1). Histologically, using the Geboes score, 41 patients (76%) had active inflammation (score ≥ 3.1), and 13 (24%) had inactive histology (score < 3.1). These findings confirm that the majority of the cohort presented ongoing mucosal inflammation as determined by the accepted gold standard methods of endoscopic and histopathological evaluation (Figure 1).

Regarding medication at the time of sampling, 23 patients (42.6%) were on 5ASA compounds, 11 (20.4%) were on biological drugs, and 14 (25.9%) were on combination therapy. Only 6 patients (11.1%) were treatment-naïve.

### 3.2. Correlation Between TNF-α Expression in Fresh and FFPE Tissues

The primary objective of this study was to determine if TNF-α mRNA quantification from routine FFPE tissues could serve as a reliable surrogate for the more complex analysis of fresh-frozen tissue. A linear regression analysis was performed comparing the TNF-α levels measured in fresh tissue (expressed as copies/ng) and the relative TNF-α expression quantified by RT-qPCR in matched FFPE samples (expressed as 2^−ΔΔCt^).

**Figure 2:**
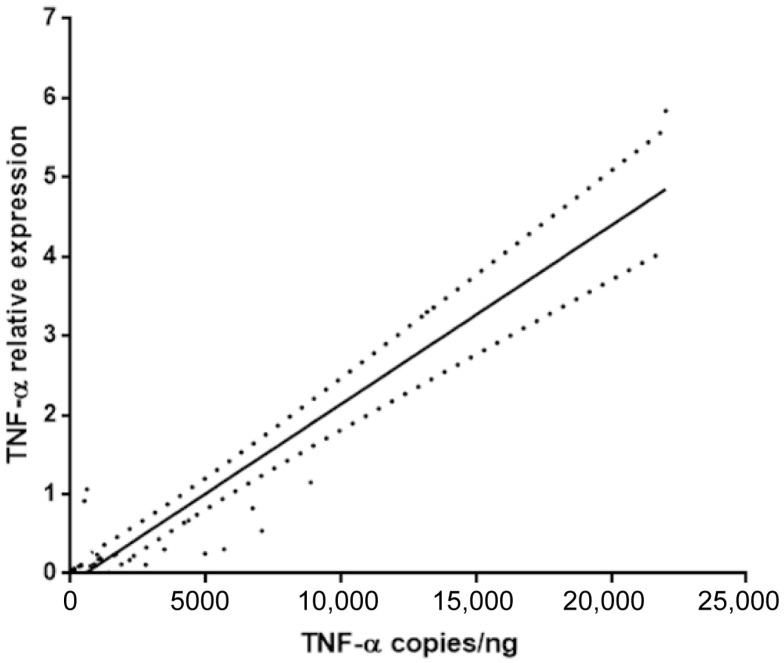
Linear regression analysis shows a significant and direct correlation between TNF-α of fresh and paraffin-embedded tissues (*p* < 0.0001; r = 0.83).

The analysis revealed a significant and strong positive correlation between the two measurement methods, as illustrated in Figure 2. The Pearson correlation coefficient was r = 0.83, indicating a very strong linear relationship. This correlation was highly statistically significant (*p* < 0.0001). This result confirms that the relative expression levels of TNF-α obtained from standard FFPE samples are highly concordant with and representative of the levels measured using the fresh tissue protocol.

### 3.3. Correlation Between Disease Activity and Biomarker Levels

The principal correlations between clinicopathological parameters and molecular markers (TNF-α measurement) are reported in Table 2.

Confirming the results of the linear regression, we found an extremely strong association between high TNF-α levels in fresh tissue of high levels in FFPE tissue. Patients with high fresh tissue TNF-α (≥3174 copies/ng) level had 114.4 times the odds of having high FFPE TNF-α expression (mean ≥ 0.67; OR = 114.4, 95% CI 12.41 to 1055; *p* < 0.0001). This finding further substantiates the interchangeability of the two methods.

Patients with active endoscopic disease (MES ≥ 1) had 28 times the odds of having high TNF-α expression in their FFPE tissue compared to patients with mucosal healing (MES < 1) (OR = 28, 95% CI 3.31 to 237; *p* < 0.0001). These findings indicate that high TNF-α levels were significantly correlated with active mucosal disease, as confirmed by both the Mayo Endoscopic Score and Geboes grading. A similar but less significant correlation was found between patients with high MES and high TNF-α level in fresh tissues (OR = 7.59, 95% CI 1.51 to 38.19; *p* < 0.012). This reinforces the strong link between molecular TNF-α expression and endoscopic inflammation.

Interestingly, patients with active histological inflammation (Geboes score ≥ 3.1) had significantly high Mayo endoscopic value <1 (OR = 4.81, 95% CI 1.26 to 18.32; *p* = 0.03).

Patients with active histological inflammation (Geboes score ≥ 3.1) had significantly higher odds of having high FFPE TNF-α levels (OR = 0.12, 95% CI 0.02 to 0.59; *p* = 0.009) and high fresh tissue TNF-α levels (OR = 7.18, 95% CI 1.69 to 30.56).

Conversely, overwear, age, sex, and pMayo score do not show a significant association with TNF-α expression in FFPE and fresh tissues.

## 4. Discussion

The overarching goal in the modern management of Inflammatory Bowel Disease is to transition from a generalized, reactive treatment paradigm to a proactive, personalized strategy that tailors therapy based on the unique biological characteristics of each patient’s disease [27]. Our study makes a substantial and practical contribution toward this goal by addressing two fundamental aspects of biomarker development. Firstly, we provide compelling evidence that archival formalin-fixed paraffin-embedded (FFPE) tissue, a universally available resource, is a reliable substrate for quantifying mucosal TNF-α mRNA. Secondly, we demonstrate that the level of this local TNF-α expression is a powerful molecular indicator that strongly correlates with both the objective endoscopic severity of Ulcerative Colitis and the histological grade evaluation.

The validation of the FFPE-based RT-qPCR method is a critical enabling step for translational research and future clinical applications. The logistical complexities, specialized infrastructure, and significant costs associated with the prospective collection and biobanking of fresh-frozen tissue have long been a bottleneck, confining tissue-based molecular studies largely to academic research centers. Our demonstration of an excellent correlation (r = 0.83; *p* < 0.0001) between TNF-α levels in FFPE and fresh tissue effectively dismantles this barrier. It confirms that the chemical processes of formalin fixation and paraffin embedding do not irreparably degrade the quantitative information held within the mRNA transcripts of key inflammatory mediators. This finding resonates with the work of James et al., who also successfully leveraged FFPE samples for TNF mRNA analysis, signaling a broader movement towards the utilization of these invaluable archival resources [15]. The implications are profound: it unlocks the potential for large-scale, cost-effective retrospective studies using existing pathology archives to discover and validate new biomarkers. More importantly, it provides a clear pathway for integrating molecular testing into the routine clinical pathology workflow. A molecular test based on the same FFPE block used for histology could provide the clinician with a composite report containing both morphological and molecular data, offering a much richer and more actionable picture of the patient’s disease state without requiring any additional invasive procedures.

Beyond methodological validation, our data provide important biological and clinical insights. Elevated mucosal TNF-α expression in FFPE samples was strongly associated with objective measures of disease activity, including endoscopic severity (Mayo Endoscopic Score ≥ 1) and histological inflammation (Geboes ≥ 3.1). In this study, endoscopic mucosal healing and histological remission, the current gold standard endpoints for disease activity in UC, were systematically assessed using the Mayo Endoscopic Score and the Geboes grading system, respectively. The strong correlation observed between TNF-α expression and both endoscopic (MES ≥ 1; OR = 28, *p* < 0.0001) and histological inflammation (Geboes ≥ 3.1; OR = 0.12, *p* = 0.009) supports the key role of TNF-α levels as one of the most important molecular markers of mucosal inflammation. Our findings are consistent with earlier studies by Olsen et al. and James et al., which demonstrated that mucosal TNF-α gene expression levels predicted therapeutic response and correlated with treatment failure, respectively [14,15,16,17]. Together, these results highlight the potential of mucosal TNF-α quantification not only as a marker of disease activity but also as a predictive biomarker for therapeutic stratification.

The clinical implications of these findings are significant. Current tools for monitoring IBD, such as CRP and fecal calprotectin, provide only indirect estimates of mucosal inflammation and are insufficient to guide precision medicine strategies. Endoscopy, while considered the gold standard, is invasive, costly, and unsuitable for repeated monitoring. By contrast, the use of FFPE tissues for TNF-α quantification offers a feasible and cost-effective alternative that can be integrated into routine pathology workflows. Importantly, this approach does not require additional biopsies beyond those already collected for histological assessment.

Our findings are also in line with previous immunohistochemical studies that demonstrated strong TNF-α expression in colonic biopsies from patients with ulcerative colitis. Olsen et al. reported high TNF-α positivity in lamina propria T cells and macrophages, with expression levels correlating with histological inflammation [17]. Similarly, Villanacci et al. observed a more diffuse and intense TNF-α immunostaining pattern in UC compared with Crohn’s disease, particularly within plasma cells, supporting its diagnostic and pathophysiological relevance [28]. Notwithstanding, the correlation between the expression of TNF-α with biologic therapy is debated and in a recent prospective study, TNF-α staining intensity was associated with non-response to biologic therapy, although differences were less pronounced among patients treated with TNF inhibitors [29].

Nevertheless, this study showed some limitations. Although the correlations were statistically robust, the retrospective nature of the study and the small sample size (*n* = 54), require confirming our data in a larger multicenter and prospective study. The cross-sectional design precludes assessment of longitudinal outcomes, such as sustained clinical remission, mucosal healing, or loss of response to anti-TNF therapy. The study focused exclusively on ulcerative colitis, and further research is warranted to determine whether similar correlations hold true in Crohn’s disease, where inflammatory patterns and histological architecture differ substantially. Another important limitation lies in the fact that, although TNF-α may represent an effective predictive marker of response to targeted therapy, in this study, we primarily focused on assessing the concordance of TNF-α expression levels between FFPE and fresh tissue samples. We plan to investigate the correlation between TNF-α expression levels and treatment response in a larger and more specific next prospective study. Taken together, our results extend the growing body of evidence supporting tissue-based molecular biomarkers in IBD and demonstrate that FFPE-derived TNF-α expression is both technically reliable and biologically relevant. By lowering logistical barriers and leveraging widely available pathology archives, this method represents a pragmatic step toward implementing personalized medicine in routine IBD care.

## 5. Conclusions

In conclusion, we validated RT-qPCR quantification of TNF-α from FFPE mucosal biopsies as a reliable surrogate for fresh tissue analysis in ulcerative colitis. High TNF-α expression in FFPE tissue correlated strongly with endoscopic and histological disease activity, underscoring its potential as a clinically meaningful biomarker. This method offers a feasible, cost-effective approach that integrates seamlessly into standard pathology workflows, providing both morphological and molecular insights from a single biopsy. Future prospective studies are warranted to evaluate the predictive value of FFPE-derived TNF-α expression for treatment response and long-term outcomes, with the goal of advancing precision medicine in IBD management.

## Figures and Tables

**Table 1:** Patients’ main clinical and biological characteristics.

	*n* = 54
Age, mean (±SD)	50 (15.35)
Age *n* (%)	
<50	26 (48.1%)
≥50	28 (51.9%)
Disease duration, years (±SD)	9.6 (±9.2)
Localization	
Proctitis	6 (11.1%)
Left-sided colitis	30 (55.6%)
Pancolitis	18 (33.3%)
Sex, *n* (%)	
Female	25 (46.2%)
Male	29 (53.8%)
pMayoS, *n* (%)	
>2	21 (38.8%)
≤2	33 (61.2%)
Mayo endoscopic *n* (%) score	
Active	39 (72.2%)
MH	15 (27.8%)
Goboes Score *n* (%)	
≥3.1	41 (76%)
<3.1	13 (24%)
TNF-α level in fresh tissue sample (mean, copies/ng)	
<3174	31 (57.4%)
≥3174	23 (42.6%)
TNF-α level in FFPE sample	
(mean, 2^−ΔΔCt^)	
>0.67	27 (50%)
≤0.67	27 (50%)
Medication at sampling	
Naïve	6 (11.1%)
5ASA	23 (42.6%)
Biological drugs	11 (20.4%)
Combination therapy	14 (25.9%)

**Table 2:** Correlation between clinical and biological parameters. The values highlighted in bold and underlined represent statistically significant results.

	Sex (M vs. F) p, OR (95% CI)	pMayo Score (≥2 vs. <2) p, OR (95% CI)	Mayo Endoscopic (≥1 vs. <1) p, OR (95% CI)	Goboes Score (≥3.1 vs. <3.1) p, OR (95% CI)	TNFa Fresh (≥3174 vs. <3174) p, OR (95% CI)	TNFa FFPE (≥0.67 vs. <0.67) p, OR (95% CI)
**Age** **(<50 vs. ≥50)**	0.5972, 0.73 (95% CI 0.25 to 2.15)	0.78, 1.32 (95% CI 0.44 to 3.95)	1. 1.086 (95% CI 0.33 to 3.58)	1. 1.11 (95% CI 0.32 to 3.88)	1. 1.023 (95% CI 0.35 to 3.01)	0.17, 0.4 (95% CI 0.13 to 1.21)
**Sex** **(M vs. F)**	\	1, 1.09 (95% CI 0.36 to 3.27)	0.36, 2.10 (95% CI 0.61 to 7.30)	0.75, 0.67 (95% CI 0.19 to 2.35)	0.27, 0.49 (95% CI 0.16 to 1.45)	0.27, 2.12 (95% CI 0.71 to 6.32)
**pMayo Score** **(≥2 vs. <2)**	\	\	0.06, 0.29 (95% CI 0.09 to 1.021)	0.32, 0.44 (95% CI 0.12 to 1.58)	0.27, 0.52 (95% CI 0.17 to 1.58)	0.26, 0.45 (95% CI 0.15 to 1.39)
**Mayo endoscopic** **(≥1 vs. <1)**	\	\	\	**0.03,** **4.81** **(95% CI 1.26 to** **18.32)**	**0.012,** **7.58** **(95% CI 1.51 to** **38.19)**	**<0.0001,** **28** **(95% CI 3.31 to 237)**
**Goboes score** **(≥3.1 vs. <3.1)**	\	\	\	\	**0.008,** **7.18** **(95% CI 1.69 to** **30.56)**	**0.009,** **0.12** **(95% CI 0.02 to** **0.59)**
**TNFa fresh** **(<3174 vs. ≥3174)**	\	\	\	\	\	**<0.0001,** **114.4** **(95% CI 12.41 to** **1055)**

## Data Availability

The data presented in this study are available on request from the corresponding author.
